# A Non-electric and Affordable Surface Engineered Particle (SEP) based Point-of-Use (POU) Water Disinfection System

**DOI:** 10.1038/s41598-019-54602-3

**Published:** 2019-12-03

**Authors:** Deepa Dixit, Virupakshi Soppina, Chinmay Ghoroi

**Affiliations:** 1 DryProTech Lab., Chemical Engineering, Indian Institute of Technology Gandhinagar, Palaj, Gandhinagar, Gujarat 382355 India; 2 Biological Engineering, Indian Institute of Technology Gandhinagar, Palaj, Gandhinagar, Gujarat 382355 India

**Keywords:** Pollution remediation, Structural properties

## Abstract

Access to safe drinking water is still a distant dream to millions of people around the world. Especially, people from the low-income group in the developing countries remain deprived of this fundamental right and causes millions of death. There is an urgent need to develop affordable and easy to handle water filter which can provide desired drinking water quality without any electricity. In the present work, a simple and low-cost surface engineered particle (SEP) based filter is developed via alkali treatment of soda-lime-silica particle. The SEP based filter can be used as a portable, non-electric, gravity-driven Point-of-Use (POU) water disinfection system. The developed SEP-based filter is capable to arrest the 99.48% (~2 to 2.5 log_10_ reduction) of gram-negative bacteria *Escherichia coli* (*E. coli* OP50) on its surface from the water containing 3 × 10^8^ cells/ml. No bacterial regrowth is observed in the purified water for 12 h. The performance of SEP bed filter is implicated to the nano-scale surface roughness, its distribution along with the surface charge and surface hydrophobicity which are favorable to attract and adhere the bacteria in the flowing water. The observation is consistent over multiple filtration cycles indicating the suitability of SEP based bed filter for POU water disinfection. The SEP surface with 0.05 mM Ag^+^ loading (SEP^+^) completely inactivated (>99.99999%) bacteria and protects any bacteria recontamination in the purified water for its long term usage. The strong and effective silver binding property of SEP surface enables very minimal silver loading and eliminates any health hazard due to low silver leaching (~50 ppb) which is well below the drinking water equivalent level (DWEL ≤ 100 ppb). In rural and urban slum areas of developing countries where no water purification system exists prior to consumption, the easy-to-implement and affordable SEP-based gravity-driven non-electric point-of-use water purifier (materials cost ~ 0.25 USD) can be used to protect millions of lives from water borne diseases.

## Introduction

Although the United Nation (UN) recognized the right to safe, acceptable, physically accessible, affordable water as a fundamental human right in 2010, more than 800 million live in the world still lacks safe drinking water at home^1^. Especially, low-income communities in rural and urban slum areas around the world drink unsafe water from wells, springs, and surface water^2^. Despite the long history of boiling as a mean to disinfect the drinking water, energy requirement limits its application in low-income communities^3^. Similarly, chemical disinfection (such as uses of chlorine) of drinking water is associated with harmful disinfection by-products (DBPs)^4^. Moreover, microbial contamination is not only related to the surface water but it can also grow in the water distribution network even after municipal water treatment in the range of 10^4^–10^5^ cells/ml^5^. Recent advancement of nanotechnology has overcome the critical problems related to water safety in developing countries. However, high-tech public health measures such as uses of nanoparticles (0D)^6^, wires or fibers (1D)^7–9^, thin films (2D), fiber assemblies (3D), and polymer gels^10,11^ are not necessarily the best. A simple, non-electric, affordable POU purifier capable of removing pathogens just prior to consumption can help millions in developing countries. Previously developed POU water purifiers incorporate doses of silver in the form of silver ions (Ag^+^) or silver nanoparticles (AgNP) on polyurethane foams^12^, fiberglass^13^, copolymer beads^14^, paper^15^, polystyrene resins beads^16,17^, alginate composite beads^18^, ceramics^19^, titania^20^, and activated carbon composite^21^. Most of the studies investigated the efficacy of silver nanoparticle against the *Escherichia coli (E. coli.)* and have shown the log-reduction value (LRV) up to 7-log reduction. However, in many cases, the contact time (between the material surface and bacteria) is much higher (~3 h) for complete inactivation (>7-log reduction). It is worthy to note that none of these silver free filter materials (i.e. uncoated) is able to reduce the bacteria number not more than 1-log reduction^22^ (~90%). Moreover, the impregnation of a very high dose of silver nanoparticles may cause potential health risk due to the release of silver in the purified water. According to the World Health Organization (WHO) guideline and the United States Environmental Protection Agency (USEPA), secondary maximum contaminant level for silver in drinking water is 0.1 mg/L (~100 ppb)^4^. Hence, there is a need for developing a cost-effective water disinfection system which has high bacteria trapping capacity without any silver or with very low loading of silver which does not have any health implication. The bacterial removal or inactivation efficiency of any filter material depends on the interaction between bacteria and the surface which can be calculated using colloid filtration theory (CFT)^23,24^ using collector efficiency (η), collision efficiency (α) and bacterial deposition rate (k_d_). While collector efficiency (η) defines the number of bacteria interacting with the surface, the probability of the bacteria sticking to the surface in the filtration system is determined with the α and k_d_. The increase in the value of α and k_d_ reflects an increase in the bacterial adhesion on the surface and vice versa.

1 $$\alpha =\frac{-2{d}_{c}\,\mathrm{ln}(1-{F}_{R})}{3(1-\theta )\eta L}$$

where L is the length of the packed bed, θ is the porosity of the medium, d_c_ is the diameter of the collector surface, and F_R_ is the fraction of bacteria that retained in the column. The collector efficiency (η) can be calculated using the Rajagopalan and Tien Model^25,26^:

2 $$\eta =4{A}_{S}^{1/3}{N}_{Pe}^{-2/3}+{A}_{S}{N}_{Lo}^{1/8}{N}_{R}^{15/8}+0.00338{A}_{s}{N}_{G}^{1.2}{N}_{R}^{-0.4}$$

where, A_s_, N_Pe_, N_Lo_, N_R_, and N_G_ are the dimensionless numbers (given in Table 1) that account for effects of neighboring particles, diffusion, London-van der Waals forces, interception, and sedimentation on particle collisions respectively^25^. The bacterial deposition rate (k_d_) is also determined using the following equation:

3 $${k}_{d}=\frac{-U}{\theta L}ln[\frac{C}{{C}_{0}}]$$

where, U is the superficial velocity, C_0,_ and C is the concentration of bacterial cells in the influent and effluent respectively.

**Table 1:** Dimensionless numbers used in collector efficiency (η) calculation^23^.

Dimensionless Number	Expression
Effect of neighbouring particle	${A}_{s}=\frac{2(1-{\gamma }^{5})}{2-3\gamma +3{\gamma }^{5}-2{\gamma }^{6}}$ where $\gamma ={(1-\theta )}^{1/3}$
Peclet Number (Ratio of convective transport to diffusive transport)	${N}_{Pe}=\frac{3\mu \pi U{d}_{c}{d}_{p}}{kT}$
London-van der Waals Force number (Ratio of interaction energy to particle’s thermal energy)	${N}_{Lo}=\frac{4H}{g\pi \mu {{d}_{p}}^{2}U}$
Dimensional number for aspect ratio	${N}_{R}=\frac{{d}_{p}}{{d}_{c}}$
Gravity Number (Ratio of stokes particle settling velocity to approach velocity of the fluid)	${N}_{G}=\frac{g({\rho }_{p}-{\rho }_{f})\times {d}_{p}^{2}}{18\times \mu \times U}$

An understanding of these mechanisms helped us to develop the new class of surface engineered particles (SEP) based non-electric POU water filter that adheres a very large number of bacteria and purifies the water. The surface texture of the SEP results in the 2–2.5 log_10_ reduction (>99%) from initial concentration 3 × 10^8^ cells/ml without using any biocides such as silver ions. These results are very significant compared to the existing literature. The very minimal loading of silver ion (Ag^+^) on the surface of SEP (called as SEP^+^) allows more than 7-log reduction (>99.99999%) of *E. coli* in the filtrate water and enable long-term storage in domestic and household drinking water application.

## Results

### Bacterial removal by SEP Filter

**Figure 1:**
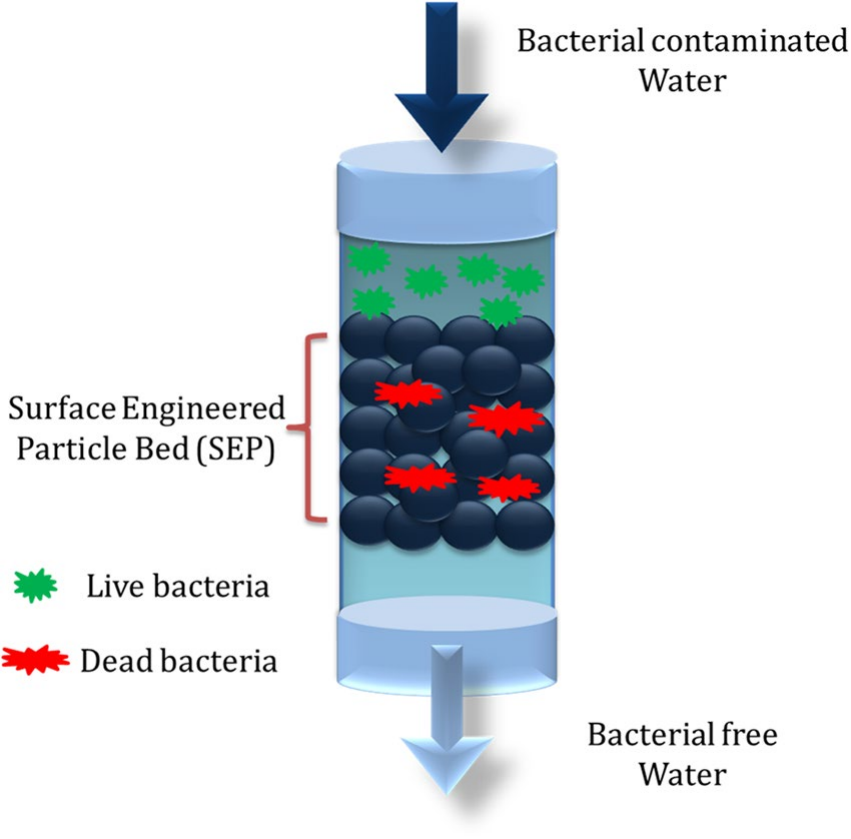
Schematic presentation of bacterial (Gram negative bacteria, *E. coli* OP50) removal filtration using surface engineered particle (SEP) bed. The influent concentration was kept ~ 3 × 10^8^ cells/ml.

**Figure 2:**
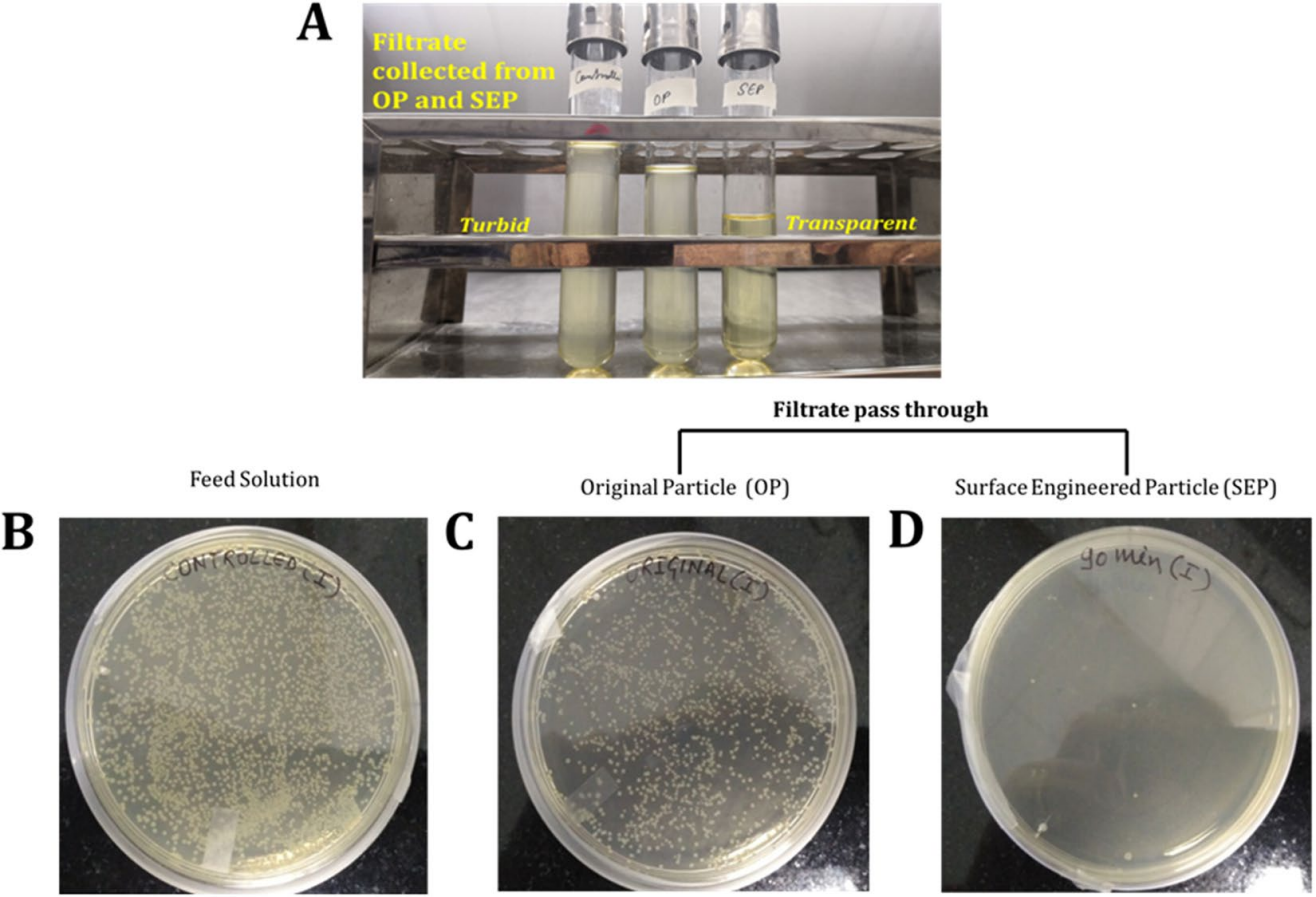
(**A**) Typical photographs of the feed solution and filtrate collected from the original particle (OP) and surface engineered particle (SEP) filter bed. While feed solution and filtrate collected from OP look similar in the turbidity, the filtrate from SEP is transparent confirming the efficacy of SEP for bacterial removal. Typical photographs of re-cultivated *E. coli* colonies on agar plate after 12 h of incubation from the (**B**) feed solution (i.e. influent), the filtrate collected from (**C**) original particle (OP) bed and (**D**) surface engineered particle (SEP) bed.

**Figure 3:**
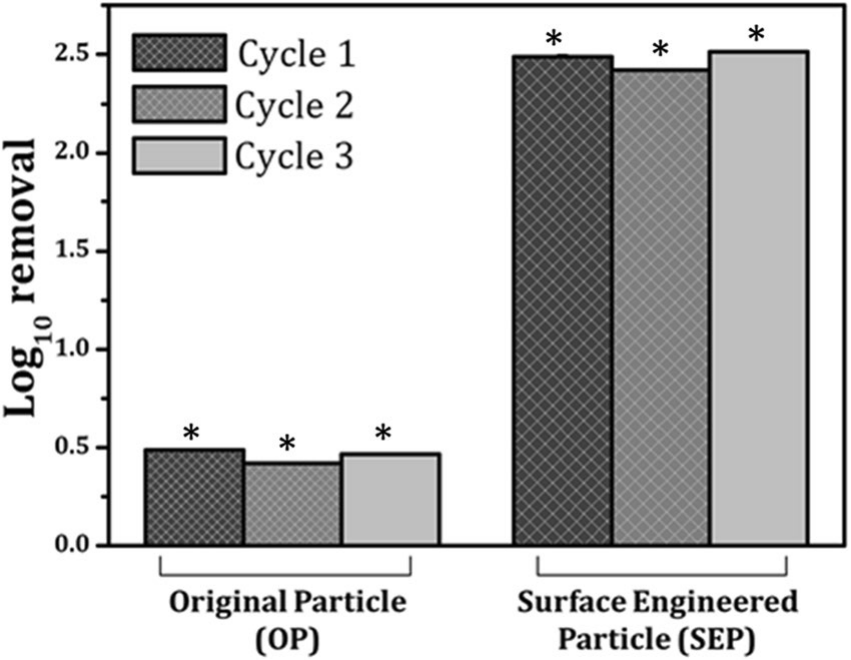
Log removal of original particle (OP) and prepared surface engineered particle (SEP) against *E. coli* OP50 showing 99.48% of the bacteria retained in SEP bed. A comparatively smaller reduction (approximately 48%) in the bacterial cells is observed with the OP samples. The performance of the SEP bed filter material was also evaluated using the cyclic test and performance found to be consistent for three cycles. Standard deviation is so small that it is not visible in the graph. Here is the *standard deviation for the presented data: OP_cycle 1: ± 0.00492, OP_cycle 2: ± 0.00730, OP_cycle 3: ± 0.00441, SEP_cycle 1: ± 0.01039, SEP_cycle 2: ± 0.00654, and SEP_cycle 3: ± 0.00718.

Experiments (schematic is shown in Fig. 1) were performed to evaluate the *E. coli* removal capacity of filter bed made up of SEP (in the absence of any Ag^+^). The SEP bed shows effective bacterial removal activity as *E. coli* suspension passed through the bed. The UV-vis spectrophotometer confirmed that 99.48% of the bacteria retained in SEP bed (equivalent to a 2–2.5 log_10_ reduction of 3 × 10^8^ cells/ml). In contrast, the original particle (OP) samples show very small log reduction (0.5 log reduction equivalent to approximately 48% reduction) in the bacterial cells (visual changes in the bacterial turbidity can be clearly seen in Fig. 2A). To further check the regrowth of bacteria in the feed solution and effluent collected from the OP and SEP bed, solutions were re-cultivated over on LB-agar plates and incubated overnight at 37 °C. Interestingly no bacterial regrowth is observed for 12 h in the agar plate with SEP which implies that a number of *E. coli* cells are not sufficient enough to regrow for 12 h after passing through SEP bed (Fig. 2D). In contrast; no difference is noticed in the growth of bacterial colonies on the feed solution and effluent collected from OP (Fig. 2B,C). Here, results provide two important information (a) the OP particle is not at all effective for bacterial removal from the contaminated water and (b) the developed SEP bed filter has remarkable bacteria trapping capacity (~2–2.5 log_10_ reduction or 99.48% bacterial removal). Therefore SEP bed reduces the number of bacteria in the effluent water efficiently and no growth of *E. coli* is noticed up to 12 h. Moreover, *E. coli* contaminated water takes an average of 10 min to pass through the SEP particle bed for complete inactivation of bacteria (approximately 3 × 10^8^ cells/ml), whereas the experiments reported in the literature generally have an exposure time to silver suspension up to 3 to 24 h for the cell concentration 10^6^–10^8^ cells/ml^22^. As POU water disinfection system does not involve long term storage of purified water, SEP-based filter without adding any silver is a good candidate for water disinfection in POU system. This indicates that the developed SEP filter is superior over many existing technologies and potential filter material for bacterial removal. The performance of the SEP bed filter material was also estimated using the cyclic test and results are found to be consistent (Fig. 3). Results confirm the potential application of SEP as non-electric POU water disinfection system for drinking water from tap or bottle or storage vessel including water distribution network where actual cell concentration is much lower than the number used in the present experiments ~ 10^8^ cells/ml.

### Bacterial Removal by SEP^+^ Filter

**Figure 4:**
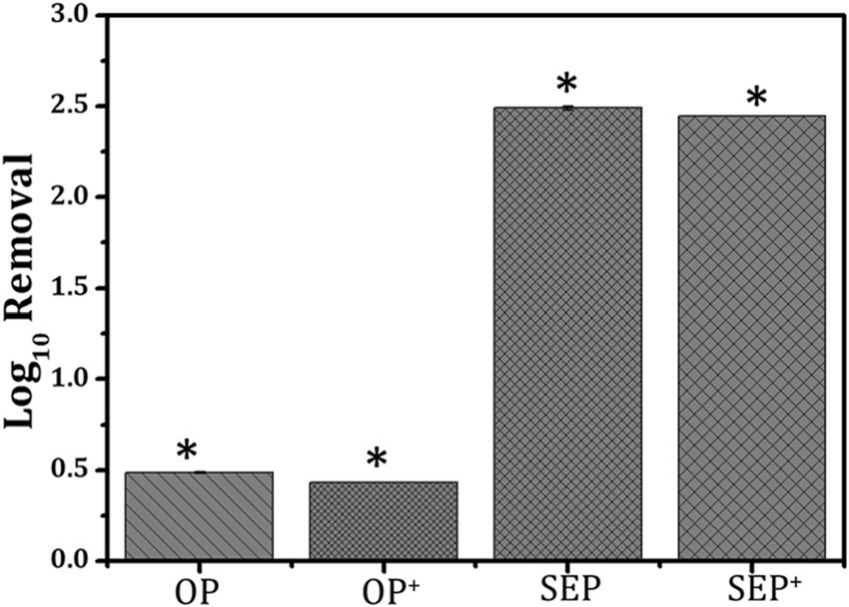
Log removal of silver loaded original (OP^+^) and surface engineered particle (SEP^+^) against *E. coli* OP50. Results show that there is no change in the bacteria removal capacity of SEP after silver loading which is very significant. The results are repeated multiple times and average value is reported. Standard deviation is so small that it is not visible in the graph. Here is the *standard deviation for the presented data: OP: ± 0.00492, OP^+^: ± 0.00256, SEP: ± 0.010390, and SEP^+^: ± 0.00478.

**Figure 5:**
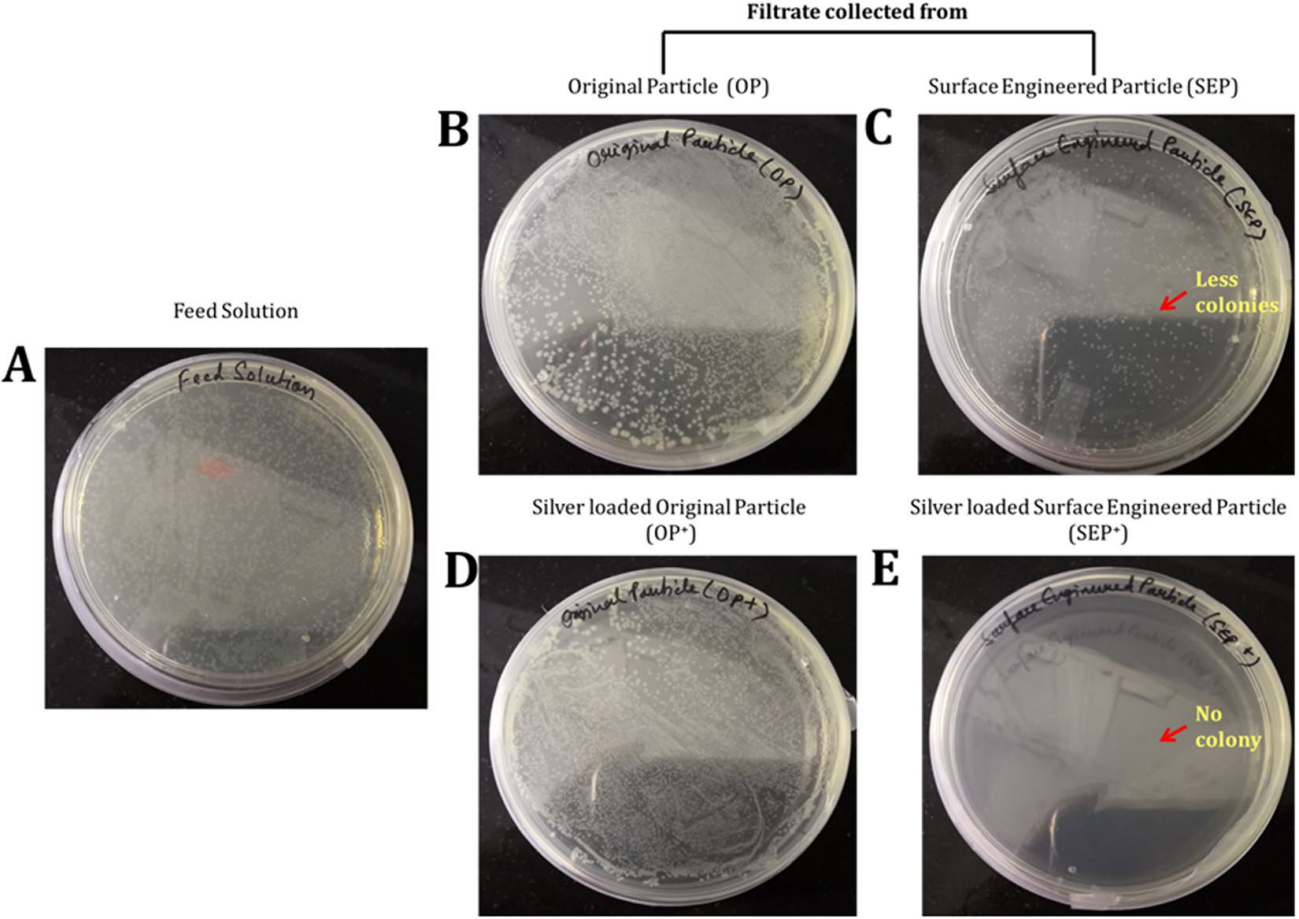
Typical photographs of re-cultivated *E. coli* colonies on agar plate after 24 h of incubation at 37 °C from the; (**A**) Feed solution, and the filtrate collected from the original particle (OP) bed and surface engineered particle (SEP) bed before (**B** and **C** in the top panel) and after (**D** and **E** in the bottom panel) silver loading respectively.

To prevent bacteria recontamination for long-term storage and usage (>12 h), further improvement in the bactericidal effectiveness of SEP bed filter was achieved using 0.05 mM AgNO_3_ (~8.49 mg/litre) loading and compared against corresponding silver loaded OP^+^ bed. Figure 4 shows the bacterial log removal in the effluent collected from OP^+^ (an original particle with Ag^+^ loading) and SEP^+^ (SEP with Ag^+^ loading) with 0.05 mM of AgNO_3_. No remarkable difference is observed in bacterial removal from particles with and without silver loading (shown in Fig. 4). The effluent collected from the OP^+^ and SEP^+^ was plated directly on the LB-agar plates and incubated for 24 h at 37 °C without any dilution. Figure 5 gives the typical photographs of *E. coli* bacteria colonies from the feed solution (Fig. 5A) and the filtrate collected from the OP and SEP before and after silver loading after 24 h of incubation at 37 °C (Fig. 5B–E). When culturing the filtrate collected from the OP and OP^+^, the regrowth of colonies can be seen clearly on the agar (Fig. 5B,D) which is similar to the feed solution (Fig. 5A). This observation implies that *E. coli* in the purified water can well survive even after passing through silver loaded OP i.e. OP^+^. On the other hand, the number of bacterial colonies on the SEP agar plate is much lesser than the feed solution and the filtrate collected from OP even after 24 h of incubation (Fig. 5C). Results attributed to the excellent bacterial removal capacity of SEP surface compare to OP surface. As can be seen from the bottom panel of Fig. 5E, there were no bacteria colonies on the agar, indicating that the *E. coli* cells cannot survive in the purified water after passing through SEP^+^. The bacteria regrowth is completely deactivated for the effluent from SEP^+^ bed. The 0.05 mM Ag^+^ loaded SEP^+^ showed no viable cells even after 24 h due to facile inactivation of bacteria. The mode of bacterial inactivation of SEP or SEP^+^ has implicated to a greater number of bacterial adhesion to the surface engineered particles. The reason for not observing any viable cell till 12 h for SEP (regrowth observed after 12 h) and after 24 h in case of SEP^+^ is implicated to the biocidal effect of Ag^+^ for SEP^+^ particles. Thus, Ag^+^ loading is found to be beneficial in three ways (a) complete removal/inactivation of bacteria which prevent the regrowth of bacteria, (b) prevent the biofilm growth^4^, and (c) reduces the number of dead bacteria in the purified water which ensures long time use of the SEP^+^ based particle filter.

To check the possible health effect, the silver leaching from SEP^+^ filter was investigated using ICP-MS analysis. The SEP^+^ filtrate is found to content ~ 50 ppb Ag^+^ which meets the USEPA and WHO standard of <100 ppb for drinking water^27^. This finding suggests that the adsorption of AgNO_3_ into the SEP^+^ is stable and indicates the general stability of the SEP^+^ filter for its potential application in water disinfection system. To the best of our knowledge, this is the first attempt to prepare and test surface engineered particles based non-electric POU water filter for bacterial removal and inactivation from contaminated water.

### SEM images of SEP and SEP^+^ inactivated Bacteria

**Figure 6:**
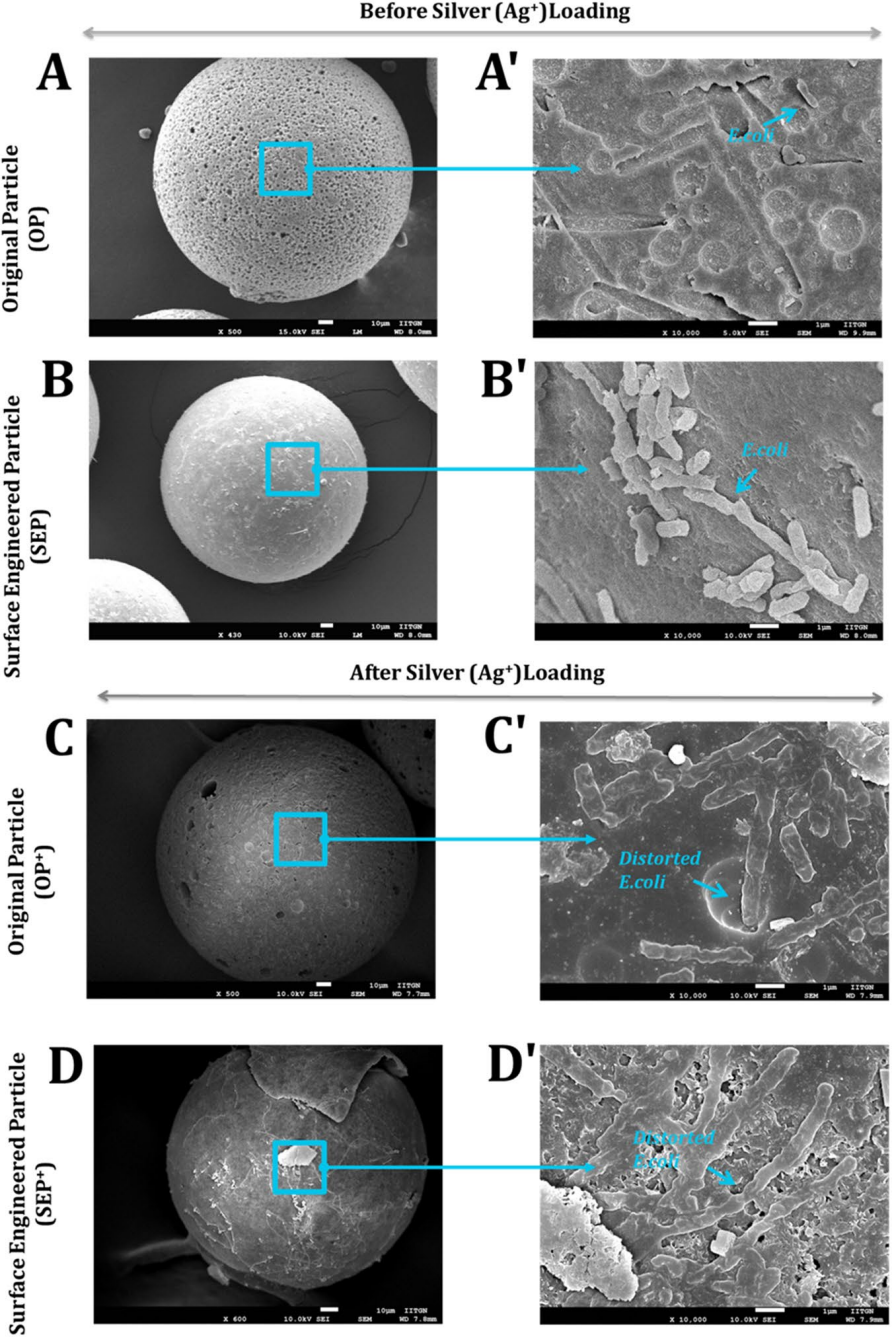
Bacterial cell morphology of *E. coli* at the surface of original particle (OP) and surface engineered particle (SEP) before (top panel) and after (bottom panel) silver loading respectively at both low (**A**–**D**) and high magnification (A′-D′). The arrows at high magnification correspond to the rectangular area at low magnification. Bacteria were fixed, dehydrated and coated with platinum (Pt) in preparation for SEM analysis. (**A**-A′) and (**B**-B′) the cells on the surface of OP and SEP before silver loading. (**C**-C′) and (**D**-D′) the cells on the surface of OP and SEP after silver loading.

The representative scanning electron microscopy (SEM) images of *E. coli* in contact with the original (OP) and surface engineered particle (SEP) before (top panel) and after (bottom panel) silver loading respectively at both low and high magnification is given in Figure 6. After washing with PBS, formaldehyde, and ethanol, no *E. coli* cells were seen on the surface of OP, however, the cells are still in intact and maintaining the expected morphology on the SEP surface (Fig. 6A′,B′). This implies that cells on the OP surface are loosely attached which results in the removal of cells after washing and hence no cells were seen on the surface of OP. The length and width of *E. coli* are measured with the software in SEM (ImageJ software). It was found that the average length and width of *E. coli* is in the range of 1.0 μm to 1.5 μm and 0.5 μm to 0.8 μm respectively. The adhered *E. coli* cells showed a discrete colonization pattern on SEP surfaces with many adhering microorganisms together (Fig. 6B,B′). However, *E. coli* cells on SEP surface show no severe morphology disruption and maintained their membrane integrity (Fig. 6B′). It is believed that bacterial adhesion on SEP surface is due to the firm grip of the cells on the regular and nanoscale surface roughness. The *E. coli* cells morphology on the surface of OP^+^ and SEP^+^ at both low and high magnification is shown in the bottom panel of Figure 6. The observation indicates that treatment with Ag^+^ induced morphological changes of *E. coli*. The length of the *E. coli* cells increases and the morphology was significantly disrupted and lost all cell membrane integrity on the surface of OP^+^ and SEP^+^ (Fig. 6C′,D′). However, the SEP^+^ surface is fully covered with the *E. coli* cells compared to the few cells on the surface of OP^+^ due to the strong bacterial adhesion capacity of the SEP followed by the biocidal effect of Ag^+^ (Fig. 6C,D). The SEM results confirm that the bacterial adhesion capacity of SEP^+^ is maintained even after silver loading. The disruption of *E. coli* membrane is due to the release of the Ag^+^^28^. Upon treatment with Ag^+^, the bacterial cells underwent considerable morphological alterations as compared to the OP and SEP.

### Surface Characterization

**Figure 7:**
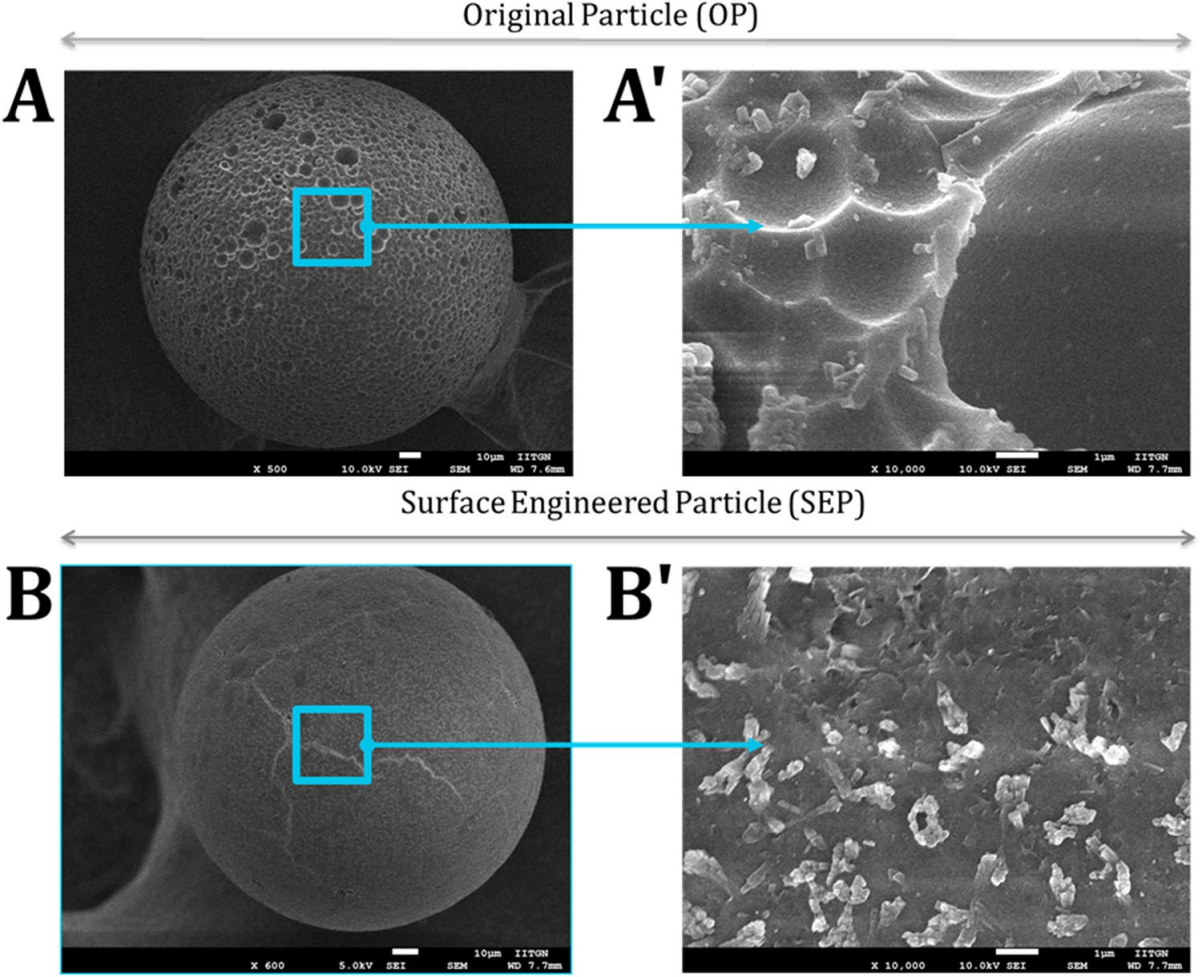
FESEM images of (**A**) original particle (OP) is showing large-scale circular clefts on the surface and (**B**) surface engineered particle (SEP) after surface treatment with the alkaline solution for 90 min. The SEP surface morphology shows the disappearance of deep cleft and material removal due to the destruction at the surface of particles with alkaline solution. A′ and B′ are higher magnification images of OP and SEP for better visualization of the surface features on the top of the microscale particle.

**Figure 8:**
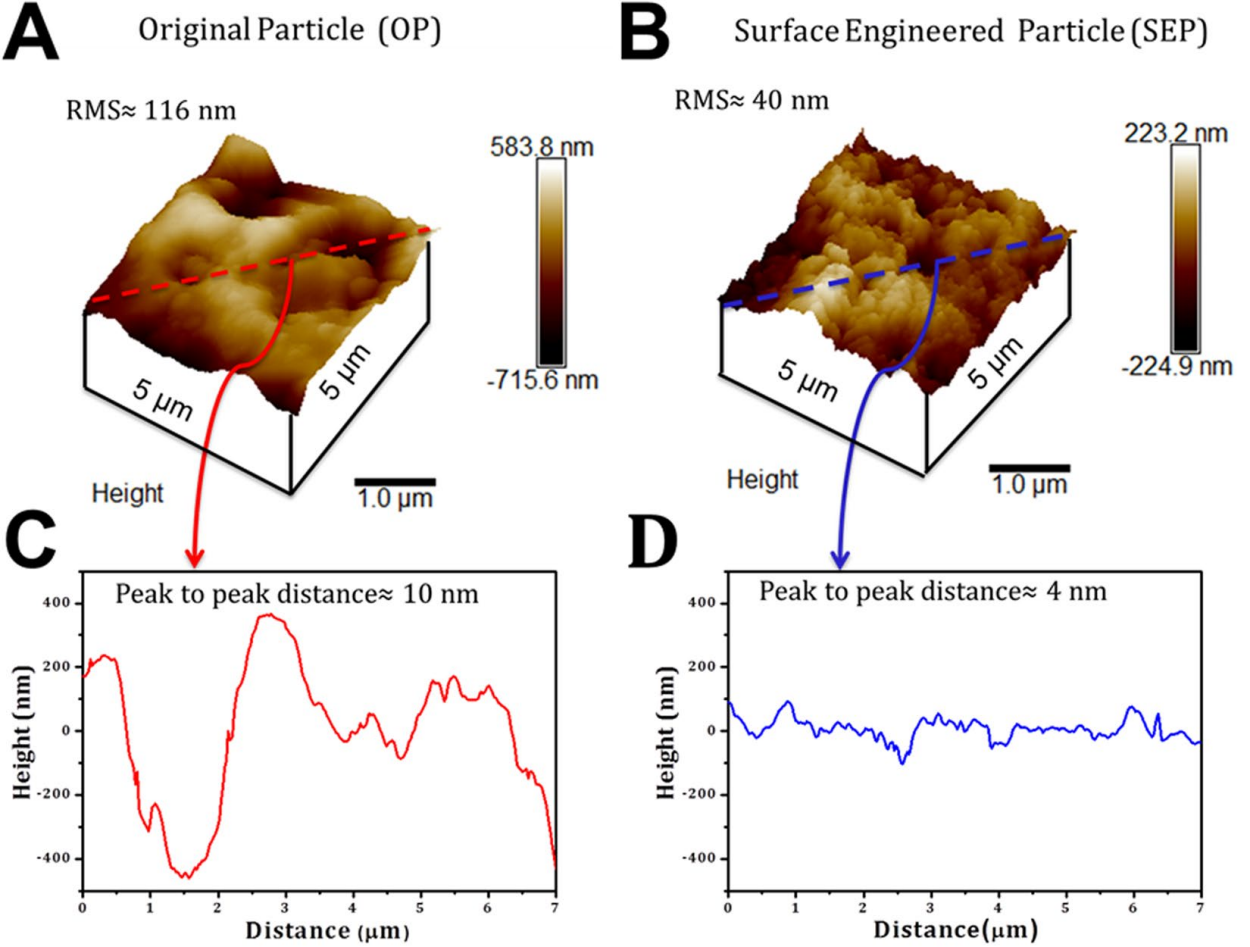
AFM images of glass particles deposited on a glass slide using double-sided tape, the surface roughness was measured on a scan size of 5 μm × 5 μm on the surface using tapping mode in the air with silicon nitride tip.: (**A**) Original particle (OP) shows the wide distribution of roughness with RMS~116 nm and average peak to peak distance found to be ~10 nm; (**B**) Surface engineered particle (SEP) shows the close distribution of roughness with RMS~40 nm approximately and average peak to peak distance ~4 nm; (**C**) surface profile for the original particle (OP) have skewness (R_skw_) greater than 0 and kurtosis (R_kur_) less than 3 showing distribution of few high peaks and low valleys; (**D**) surface profile for the surface engineered particle (SEP) showing showed an R_skw_ close to 0 and R_kur_ greater than 3 which means SEP exhibits distribution of many high peak and low valleys.

Figure 7 shows the SEM micrograph of the OP and SEP particles. SEM images of OP and SEP glass particles reveal that the particles are spherical in shape (shown in Fig. 7A,B). Large-scale circular clefts are observed on the surface of OP (shown in Fig. 7A,A′). Surface modification with alkaline medium leads to the disappearance of deep cleft and material removal due to the destruction at the surface of glass particles is clearly observed at both low and high magnification respectively (Fig. 7B,B′)). A cleaner surface with comparative tiny structures is observed at the surface of SEP. The tiny structures on the surface of SEP form a uniformly distributed rough surface. This observation is further corroborated with Atomic Force Microscopy topographical images shown in Figure 8.

The quantitative values of surface topographical features from AFM measurement are enumerated in Table 2. Five parameters were used for the characterization of the surface; (a) Root Mean Square (RMS) roughness (R_q_), (b) peak to peak distance, (c) skewness (R_skw_), (d) kurtosis (R_kur_), and (e) aspect ratio. While R_q_ and peak to peak distance were used to define the changes in surface topography of the SEP, skewness, and kurtosis were used to describe the surface morphology^29^. The AFM measurement data shows that the surface modification reduces the RMS roughness to approximately one third and peak to peak distance to half of the OP particles. The SEP glass particle surface showed an R_skw_ close to 0 and R_kur_ greater than 3, indicates that the SEP surface exhibited a distribution of many high peaks and low valleys compared to the exhibited distribution of few high peaks and low valleys on OP surface (Table 2). In addition, the aspect ratio of SEP is smaller than the OP particle surface. The surface modification with alkaline medium produces nano-scale asperities at the surface of SEP (the tiny structures seen in SEM). The minimum surface roughness and peak to peak distance reduce from 116 nm to 41 nm and 9.72 nm to 4.67 nm respectively. Figure 8A represents the surface topography of OP particle showing that surface covered with few structures of large height and width. Whereas the destruction of the big cleft and many peaks and valleys can be clearly seen at the surface of SEP which is attributed to erosion at the surface due to alkaline treatment, shown in Figure 8B.

**Table 2:** RMS roughness, average roughness, peak to peak distance, skewness, kurtosis and aspect ratio for the original and surface engineered particles obtained using Atomic Force Microscopy (AFM)

Samples	RMS, R_q_ (nm)	Peak to peak distance (nm)	Skewness, (R_skw_)	Kurtosis R_kur_	Aspect Ratio
OP	116 ± 24.54	9.72	−0.15	3.58	2.04
SEP	41 ± 8.31	4.67	−0.43	6.90	1.66

OP- Original Particle, SEP- Surface Engineered Particle.

Section analysis in AFM is used to obtain the surface profile of OP and SEP particles (shown in Fig. 8C,D). Results clearly indicate the local changes in the peak height (positive and negative) and peak to peak distance after surface modification. The roughness distribution at the surface of SEP particles became narrower and more uniform compared to OP (shown in Fig. 8D). These observations signify that the scratched and chipped parts of the surface removed and the new surface is created. The change in surface profile of SEP may result in increase in the (i) number of contact points, (ii) incorporation of new chemical functional sites, and (iii) more turbulence near the surface of the SEP during water flow^30^.

**Figure 9:**
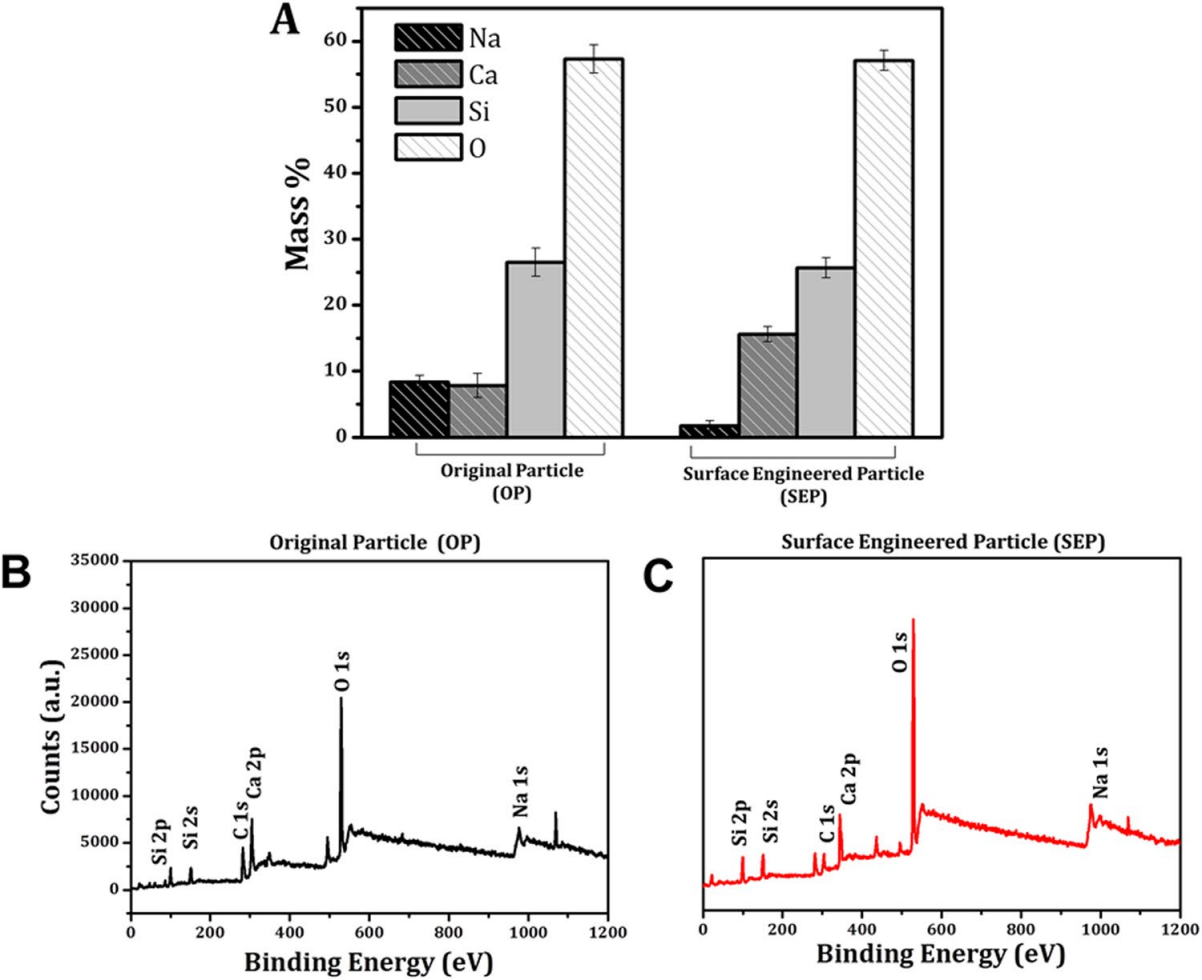
(**A**) Surface elemental composition from XPS results – indicating an increase and decrease in calcium and sodium percentage respectively on the surface of SEP particles, (**B,C**) are the wide scan spectra for the original particle (OP) and Surface engineered particle (SEP) respectively. XPS survey scan measured on OP and SEP particles showed five peaks at binding energy of about 103.5 eV, 284.6 eV, 347.3 eV, 533.3 eV, and 1071.0 eV attributed to the Si 2p, C 1s, Ca 2p, O 1s, and Na 1s.

**Figure 10:**
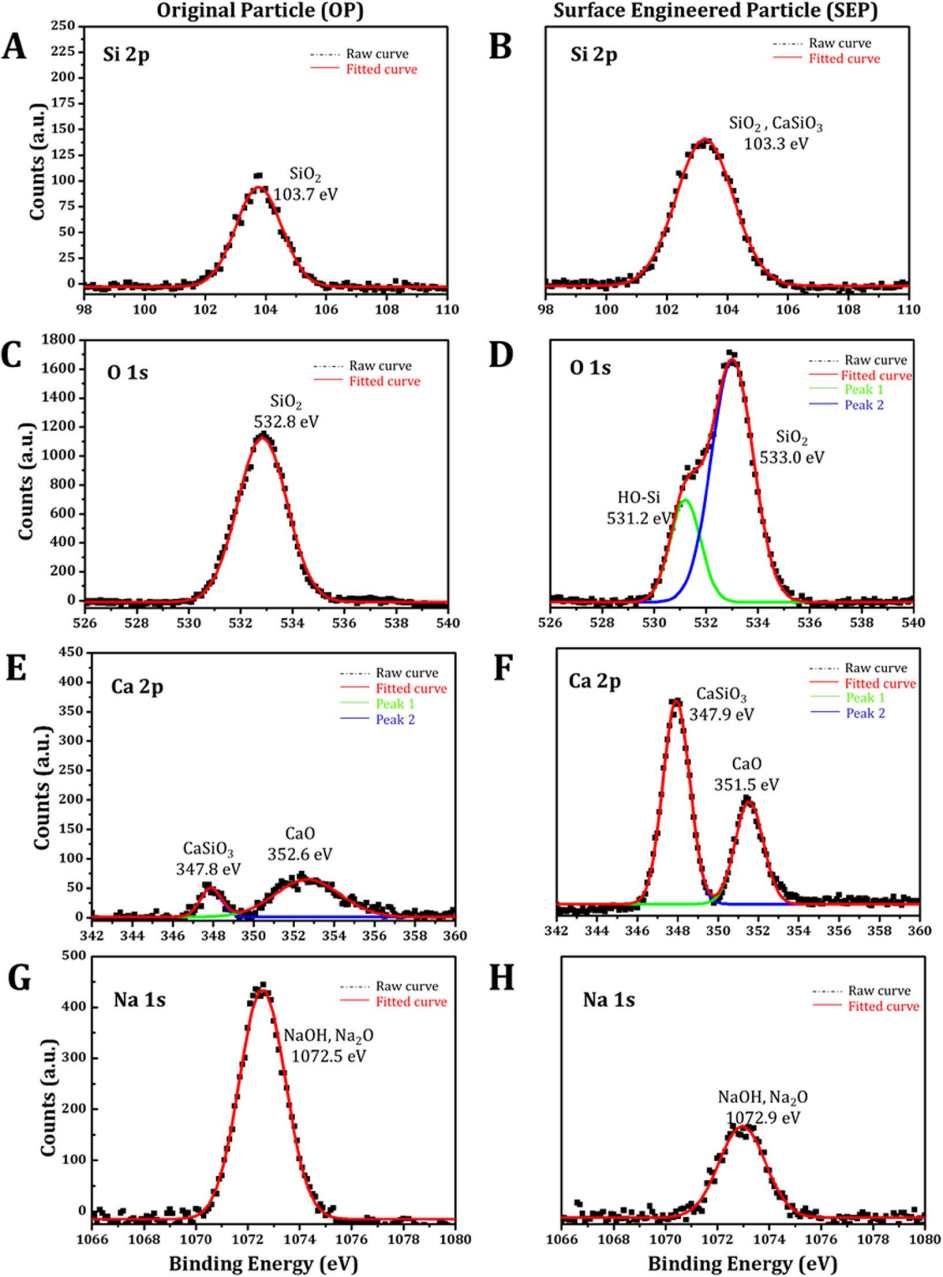
XPS studies to determine the chemical functionality at the surface of original and surface engineered particle. The spectra are fitted with Gaussian distribution. (**A)** Si 2p high-resolution spectra for the original particle, spectra shows the broad peak at 103.7 eV which is attributed to the SiO_2_; (**B**) Si 2p high-resolution spectra for surface engineered particle (SEP), spectra exhibits a single peak at 103.3 eV which is attributed to the SiO_2_ and CaSiO_3_ formation at the surface due to chemical reaction of glass particles in alkaline medium; (**C**) O 1s high-resolution spectra for the original particle with single peak which is resulted due to SiO_2_ at the surface; (**D**) O 1s high-resolution spectra for the surface engineered particle showing two peaks: peak 1 at 531.2 eV corresponds to the OH group attached to the silica (Si-OH) at and peak 2 at 533.0 eV shows that the oxygen is also present in the form of SiO_2_ at the surface; (**E**) Ca 2p high-resolution spectra for the original particle showing two peaks: peak 1 at 347.8 eV is attributed to the CaSiO_3_ and the peak 2 at 352.6 eV confirms the presence of CaO at the surface of original particle; (**F**) Ca 2p high-resolution spectra for the surface engineered particle (SEP) showing two peaks: sharp peak 1 at 347.9 eV confirms the formation of CaSiO_3_ at the surface and smaller peak at 351.5 eV corresponds to the CaO at the surface. (**G,H**) Na 1s high-resolution spectra for the original and surface engineered particle exhibits peak at 1072.5 eV and 1072.9 eV respectively attributed to the NaOH and Na_2_O at the surface.

To check the surface chemical changes, XPS survey scan was measured on OP and SEP particles showed five peaks at binding energy of about 103.5 eV, 284.6 eV, 347.3 eV, 533.3 eV, and 1071.0 eV attributed to the Si 2p, C 1s, Ca 2p, O 1s, and Na 1s (shown in Fig. 9A–C). The elemental composition at the surface of OP and SEP is shown in Figure 9A. These results confirm the elemental composition of soda lime silica glass particles. While the percentage composition of Si and O is the same at the surface of OP and SEP, the percentage composition of divalent cation Ca has increased on the surface of SEP compared to the OP. For comparative analysis, high-resolution XPS spectra obtained for Si 2p, O 1s, Ca 2p and Na 1s of OP and SEP (shown in Fig. 10A–H). The deconvoluted Si 2p spectra for OP showed a broad peak appeared at 103.7 eV is ascribed O-Si-O (shown in Fig. 10A). The Si 2p peak appeared at 103.3 eV towards lower binding energy is ascribed to the Si-OH due to the formation of –OH groups and formation of CaSiO_3_ at the surface of SEP due to the reaction with alkaline medium (Fig. 10B). The O 1s high-resolution spectra for OP and SEP glass particles are shown in Figure 10C,D. There is a significant compositional dependence of the spectra. The peak at 532.8 eV is attributed to the SiO_2_ groups (bridging oxygen) at the surface of OP glass particles. The deconvoluted O 1s spectra for SEP glass particles show a broad peak related to O atom, which can be further deconvoluted into two peaks as follows: the peaks appeared at 531.2 eV and 533.0 eV are respectively attributed to Si-OH and SiO_2_ (Fig. 10D). The lower binding energy peak at 531.2 eV corresponds to the non-bridging oxygen which occurs due to the formation Si-OH due to alkaline treatment. The deconvulated Ca 2p spectra for the OP and SEP exhibit two peaks respectively (shown in Fig. 10E,F). While peak 1 at 347.8 eV attributed to the CaSiO_3_ which is due to the reaction of CaO with the SiO_2_ and peak 2 at 352.6 eV confirms the presence of CaO at the surface of the OP. The area under the peak 1 at 347.9 eV for the SEP is much higher for the CaSiO_3_ due to the reaction in alkaline medium. In contrast, the area under the peak of Na 1s decreases for SEP as shown in Figure 10G,H. The relative peak area due to Si-OH, CaSiO_3_, O-Si-O, CaO groups before and after alkaline treatment is compared in Table 3. The alkali treatment results in higher percentage Si-OH and CaSiO_3_ on the SEP surface compared to the OP surface.

**Table 3:** Comparison of relative peak area (%) of O 1s, Si 2p, Ca 2p, and Na 1s obtained from high-resolution XPS spectra.

Sample	SiO_2_	SiOH	CaO	CaSiO_3_	Na_2_O
OP	67.21	0	6.38	1.60	24.81
SEP	57.89	17.98	6.09	11.44	6.59

OP- Original Particle, SEP- Surface Engineered Particle.

**Figure 11:**
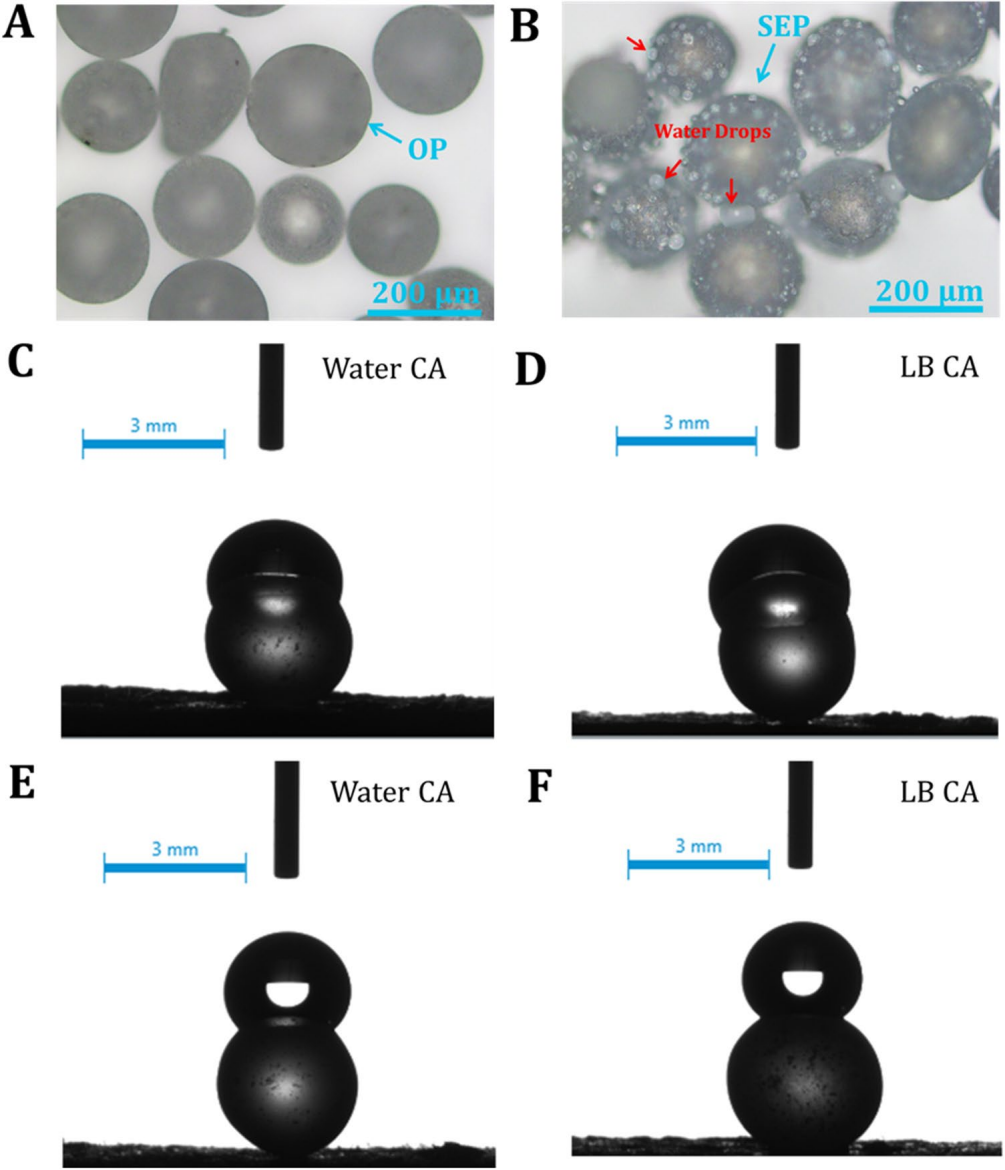
Optical microscope images; (**A**) Original particle (OP) and (**B**) Surface engineered particle (SEP) when dispersed in water. Red arrow in B representing the water drops at the SEP surface. (**C,D**) showing the water and liquid LB media drop at the surface of OP respectively, (**E,F**) showing the water and liquid LB media drop at the surface of SEP respectively. The SEP has a much higher contact angle for both water and LB media than OP.

As the static contact angle (CA) measurement is difficult on the surface of 150–200 μm size particles, the wetting nature of the OP and SEP was observed using optical microscope (Fig. 11A,B) after dispersing water on to the particles. As can be seen from the images, the surface and water interaction of SEP surface is completely different from the OP surface. While the OP surface shows a continuous film type wetting, the water sits in the form of tiny droplets (red arrows in Fig. 11B) on the SEP surface. The observation indicates the SEP surface is partially wetting in contrast to the OP surface which is fully wetting. To quantify the varying wetting nature of OP and SEP, the separate static CA measurement with water and LB media using 2–3 mm particles with the same composition (contact angle with LB media see in supporting information Movie S1 and S2). The water drop shape (volume 1 μl) at the surface of OP and SEP glass particle is shown in Figure 11(C–F). The OP glass particles showed a hydrophilic contact angle of 79° (average) ± 0.07 (standard deviation) for both water and LB. In contrast, the contact angle on the surface of SEP glass particles is increased from 79° to ~147° ± 2.76 for both water and LB media. These results confirm the change in wetting behavior from hydrophilic OP to the highly hydrophobic nature of SEP glass particles. Secondly, the LB and water interact with both surfaces in a similar way. The higher contact angle (CA) for the SEP signifies the role of surface features in improving the CA beyond 120° which is very well stated in the previous studies^31,32^. The high contact angle at the surface of SEP glass particles is mainly due to the surface topography and nanoscale surface roughness shown in SEM and AFM analysis. As discussed earlier, root mean square roughness (R_rms_) of SEP is much smaller than the OP. Also, the spacing between the pillars decreased from ~10 nm to ~5 nm which leads to high contact angle (~147°) at the surface of SEP glass particles. These results demonstrate that the addition of nano-scale surface roughness changes the wetting behavior of surface from the hydrophilic to a highly hydrophobic.

**Figure 12:**
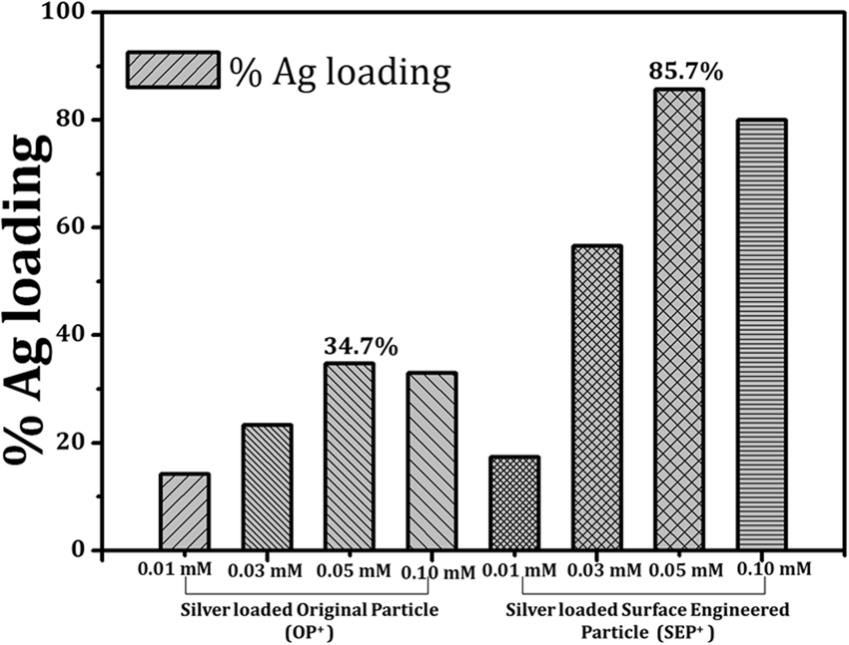
Percentage loading of AgNO_3_ on the surface of OP and SEP particles for different concentration of AgNO_3_ i.e. 0.01 mM, 0.03 mM, 0.05 mM and 0.10 mM. The error bars are standard deviation from triplicate measurements. The Ag^+^ loading found to be much higher for SEP than OP. Experimental conditions: an acid digestion was performed of the supernatant collected from the loading experiments and analysed the amount of silver with Inductive Coupled Plasma Optical Emission Spectrometry (ICP-OES, GF-AA, Perkin- Elmer Analyst 100).

The percentage loading of Ag^+^ on the surface of OP and SEP was measured using ICP-OES after complete digestion with HNO_3_ and H_2_O_2_. The actual % of Ag^+^ loading vs. concentration of AgNO_3_ is shown in Figure 12. The percentage of Ag^+^ loading increases with increasing the AgNO_3_ concentration and then decreases for both OP and SEP. The highest loading was observed for 0.05 mM concentration of AgNO_3_ in the SEP (85.7% for SEP against 34.7% for OP). The OP has OH^-^ and the density of OH^−^ ions increases when it is treated with the NaOH solution which is confirmed with XPS studies. This is why the Ag^+^ percentage loading for the SEP is more effective than the OP glass particles (shown in Fig. 12). Due to the highest loading of Ag^+^, bactericidal behaviour of OP^+^ and SEP^+^ was studied only for the particles treated with 0.05 mM AgNO_3_. The actual Ag^+^ loading on the OP^+^ surface and SEP^+^ surfaces are 0.017 mM and 0.042 mM Ag^+^ respectively.

## Discussions

### Bacterial removal/inactivation mechanism

**Figure 13:**
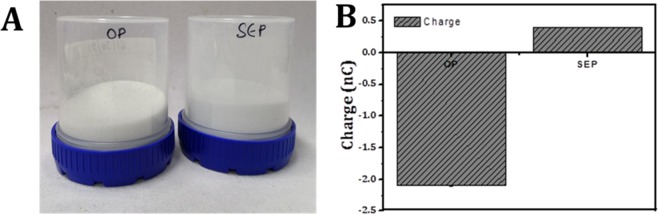
(**A**) Stick test and (**B**) Faraday cup test was performed to quickly determine the charge characteristic properties of the Original particle (OP) and Surface engineered particle (SEP). Positively charged SEP particles stuck to the surface of negatively charged plastic container compared to the original particle showing no sticking effect.

The bacterial cell adhesion mechanism on any surface dictates by both reversible and irreversible processes. In the reversible process, (temporary attachment) the cells attach to the surface due to electrostatic and hydrodynamic interaction (non-specific interaction)^33,34^. In contrast, in the irreversible phase (permanent attachment) bacteria interact with the surface due to van der Waal forces and hydrophobic interaction (i.e. specific interaction). Hence, the bacterial removal and inactivation by SEP may occur in two stages; firstly the bacteria come closer to the surface by electrostatic attraction and hydrodynamic interaction. Secondly, it attaches to the surface of SEP due to the van der Waal forces and hydrophobic interaction at the favourable sites. The first step happened due to a higher concentration of divalent cation Ca^2+^ at the surface of SEP along with the hydrodynamic interaction (Refer XPS results Fig. 10E,F)^35^. The negatively charged bacteria attract more towards to the positively charged SEP surface compared to the OP^35^. In addition to XPS, a stick test^36^ (visual observation) and Faraday cup test^37^ (quantification) were performed to depict the electrostatic charge at the SEP surface. Positively charged SEP particles stuck to the surface of negatively charged plastic container compared to the original particle showing no sticking effect (Fig. 13A). Similarly, in the Faraday cup test, the SEP particle showed approximately + 0.5 nC compared to the OP which has −2.0 nC charge on its surface (Fig. 13B). This strongly suggests a favorable deposition condition for negatively charged bacteria on SEP surface. Secondly, the surface features of the SEP (RMS~ 41 nm, peak to peak distance~ 4.67 nm) is in the order of the morphology of outer side membrane of *E. coli* (Pilli length~2–20 nm, Pilli diameter ~ 8.5–9.5 nm, and flagella diameter~ 20 nm)^38^ which can accommodate more number of *E. coli* than OP due to narrow distribution of nano-scale roughness on the surface of SEP. In addition, the many high peak and low valleys at the surface of SEP increases the access of bacterial cells to the nano-size spaces and achieve a better grip at the surface (Fig. 8D). The tight contact due to strong adhesion between the cells and the SEP surface protects the cells from the external shear forces. The retention of bacterial cells on SEP surface even after rigorous washing (refer Fig. 6B,B′) confirms the strong binding potential of the SEP surface. This implies the stronger adhesion and protection from the environment (mainly due to shear forces) of *E. coli*. on the surface of SEP is due to the more contact points and enhanced surface area (OP = 0.3801 m^2^/gm, SEP = 1.8083 m^2^/gm) which is not the case with OP surface.

In addition, the non-wetting nature of the SEP particles played an important role in the effective bacterial removal capacity compared to OP. Figure 11 showed that the SEP surface is hydrophobic compared to the hydrophilic OP surface. This helps to increase the non-specific interaction with the bacterial cell membrane. Therefore, when media passes through the hydrophobic SEP filter, bacteria gets adhere/adsorbed on the surface leaving behind the liquid medium. Hence bacteria prefer to retain at the SEP surface than moving along with the flow. Once the bacterial cells strengthen the grip at the SEP surface, the high density of reactive oxygen species OH groups at the SEP surface (refer Fig. 10C,D) deactivate the activity of the cells at the surface^25^ which reflects in the no colony formation on the agar plate up to 12 h (refer Fig. 2). However, bacteria start growing after 12 h on the agar but the number of bacterial colonies is much lesser than the OP (Fig. 5B,C).

**Figure 14:**
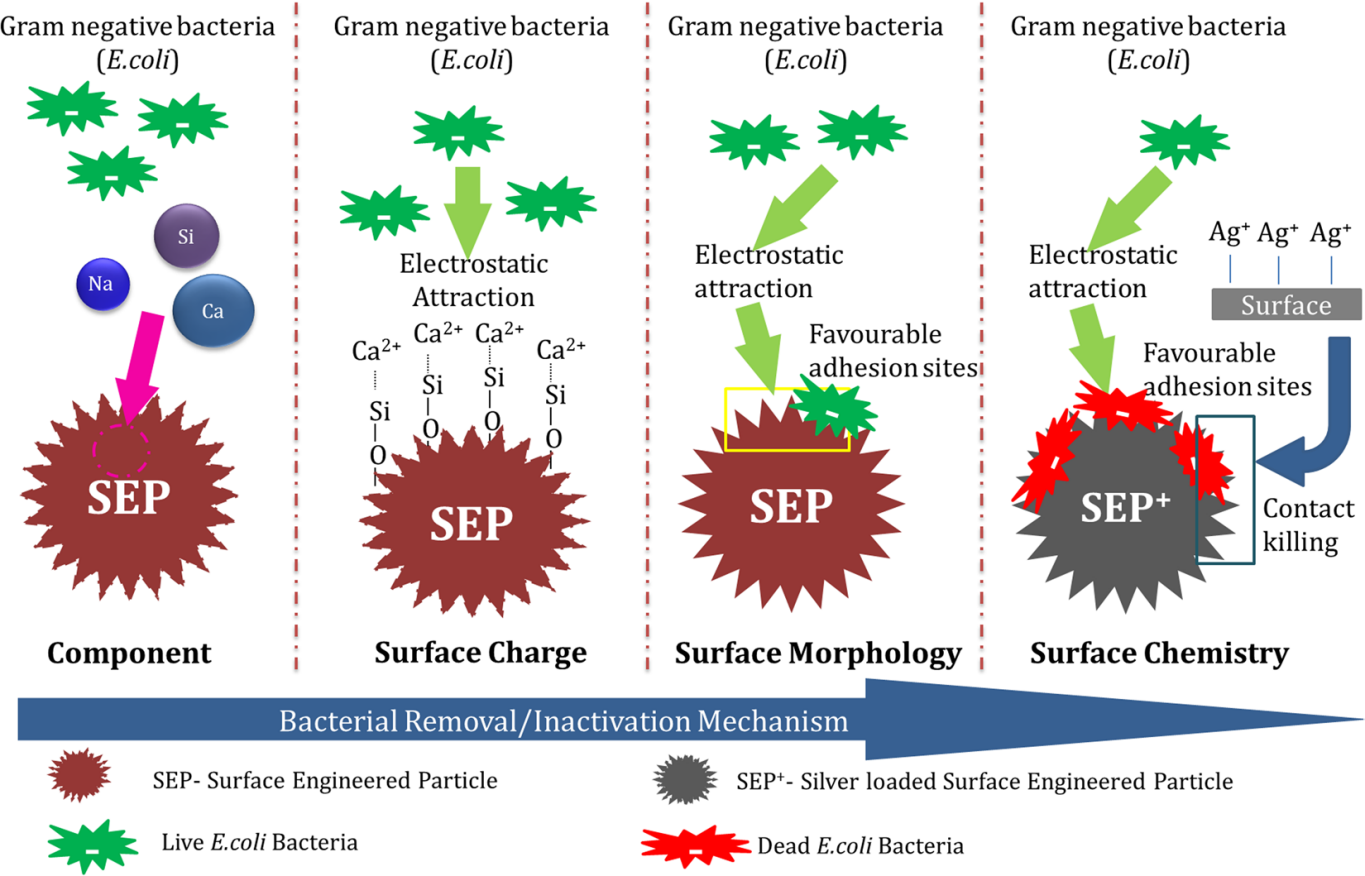
Illustration depicting the bacterial disinfection mechanism of surface engineered particle (SEP) and silver loaded surface engineered particle (SEP^+^) in bacteria-contaminated water.

For complete inactivation of cells in the purified water for long term storage and usage SEP^+^ is used. The bacterial inactivation mechanism is further enhanced for the SEP^+^ due to contact killing by Ag^+^. The mode of bacterial inactivation of SEP^+^ can be correlated with the greater bacterial adhesion capacity of the SEP surface. The key difference in the bactericidal activity of the OP^+^ and SEP^+^ includes the change in surface features, hydrophobic nature, and effective silver loading. As, the percentage silver loading of 0.05 mM AgNO_3_ in the OP^+^ i.e. 34.7% (shown in Fig. 12) which is much less than SEP^+^ (approximately 85.7%). The inactivation of bacteria believes to happen in the two stages; Firstly the bacteria attach to the surface of SEP^+^ due to the electrostatic attraction, hydrophobic interaction and favourable adhesion sites (as discussed above) which is followed by contact killing by silver ions at the sites (Schematic depicting bacterial disinfection mechanism of SEP and SEP^+^ is shown in Fig. 14).

**Figure 15:**
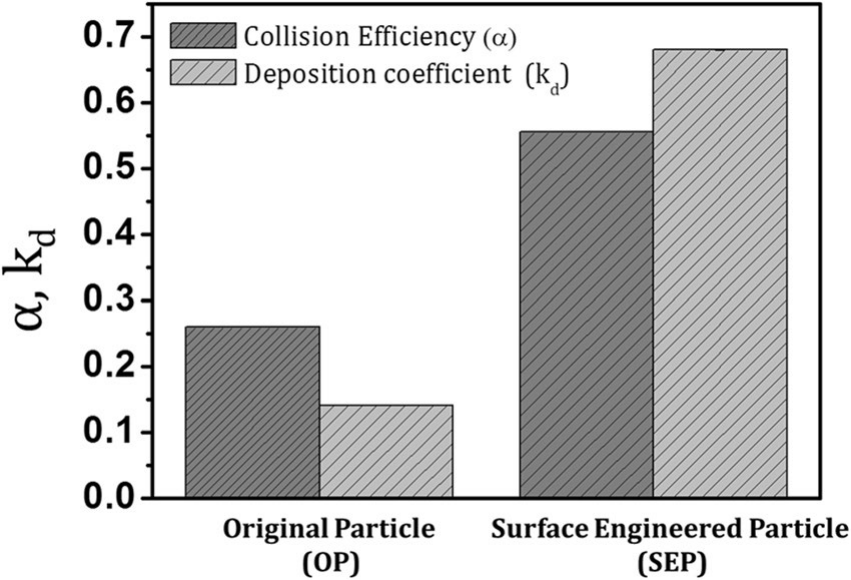
Collision efficiency (α) and deposition coefficient (k_d_) for bacterial cells measured using CFT with original particle (OP) and surface engineered particle (SEP).

The CFT model calculation for collision efficiency (α) and deposition coefficient (k_d_) also confirms the increased bacterial adhesion on the SEP surface (Fig. 15). The dimensionless numbers and parameters used in collector efficiency (η), collision efficiency (α), and deposition coefficient (k_d_) calculation is given in Table 1 and Table 4 respectively. Though the collector efficiency (η) of the OP and SEP is approximately same (in the range of 0.0066–0.0088), the collision efficiency and deposition coefficient of SEP surface (α_SEP_ = 0.5552 ± 0.0012, k_d_ = 0.68 ± 0.0015) are double than that of the OP surface (α_OP_ = 0.2598 ± 0.0014, k_d_ = 0.14 ± 0.0013). In fact, the deposition coefficient (k_d_) increased about 4.8 times for SEP particles indicating superior bacterial adhesion capability of the SEP surface. Results confirmed that engineering the surface roughness and surface chemistry of the material can inactivate and remove the bacteria effectively in the POU water disinfection system. Moreover, it increases the effectiveness of silver binding which enables the very minimal amount of silver loading for the complete killing of bacteria in the contaminated water. Thus, it can reduce the overall cost of the SEP-based filter. In fact, the cost of the developed material is ~ 0.25 USD which is much less than the existing products.

**Table 4:** Parameters used in collision efficiency (α) calculation.

Symbols	Parameters	OP	SEP
L	Length of the pecked bed (m)**	0.10	0.10
θ	Porosity of the medium**	0.35	0.35
d_c_	Collector diameter (m)**	0.00015 ± 3.65	0.00012 ± 5.15
F_r_	Fraction of particles that retained in the column**	0.4800 ± 0.00492	0.995 ± 0.01039
k	Boltzman constant (kg.m^2^/s^2^.K)*	1.38E-23	1.38E-23
T	Fluid temperature (K)	298	298
U	Superficial velocity (m/s)**	0.00586	0.00586
d_p_	Equivalent diameter of the bacterium (m)**	0.0000015	0.0000015
μ	Dynamic fluid Viscosity (Ns/m^2^)*	0.00089	0.00089
ρ_p_	Density of particle (kg/m^3^)**	2.5	2.5
ρ_f_	Fluid density (kg/m^3^)*	997.1	997.1
g	Acceleration due to gravity (m/s^2^)*	9.81	9.81
H	Assumed Hamaker constant (Joule)*	1E-20	1E-20

OP- Original Particle, SEP- Surface Engineered Particle, *^23^, and ** Experimentally determined.

## Conclusion

In this work, a surface engineered particle (SEP) based point-of-use (POU) water filter prepared via the alkali treatment of soda lime silica glass particles. The prepared SEP-based low cost and non-electric filter showed high removal and inactivation efficiency of bacterial (*E. coli*) contamination. The SEP-based filter can remove the bacteria up to 2–3 log-reduction from 3 × 10^8^ cells/ml without using any silver Moreover, purified water can be stored for 12 hours without any regrowth for point-of-use application. The present SEP material removes and inactivates the pathogens from contaminated water by coupling the nanoscale surface roughness with the surface charge and surface chemistry. Moreover, highly hydrophobic nature enhances bacterial interaction with the surface. The addition of Ag^+^ on SEP surface further enhances the bacterial inactivation efficiency by contact killing of bacteria in SEP^+^ based filter. In addition, the regulate leaching of Ag^+^ from the rough surface of SEP^+^ prevents recontamination in the purified water for long term storage and usage. The technology is simple, low-cost, portable, non-electric, gravity-driven and can be adapted to household use or community use including situations like a natural disaster. Therefore, the developed technology has bacterial removal potential and can save millions of people from water-borne diseases.

## Methods

### Materials

The water filter was made up of soda lime silica particles (supplied by Indo Glass Particles, India) treated with the alkaline solution (4 M NaOH, purchased from SRL, India) for point-of-use water disinfection. The diameter of the glass particles is in the range of 150–160 µm and density in the range 2.5–2.55 gm/cm^3^, and was free from any chemical additives. Silver nitrate (AgNO_3_), 30% hydrogen peroxide (H_2_O_2_), 69% nitric acid (HNO_3_), formaldehyde and ethanol were purchased from Sigma Aldrich and used as received. Nutrient broth and agar (Luria-Bertani) were purchased from the Hi-Media India. Acetone and Isopropanol supplied by SRL, India were used for washing. Deionized water (Merck Millipore, India, 18.2 MΩ) was used throughout the experiments. All culture media and containers for bacteria study were autoclaved at 121 °C for 45 mins and sterilized glass wool was used for glass particles packing in the column water disinfection experiment.

### Surface characterization methods

#### Scanning electron microscopy

The Scanning Electron Microscopy (JEOL JSM 7600 F, USA) is used to visually assess the particle shape and morphology of the OP, SEP particles. Atomic Force Microscopy**:** In order to quantify the changes in surface topography and surface profiles, the OP and SEP particles surfaces were analyzed via Atomic Force Microscopy (AFM) using multimode Nanoscope8 (Bruker-AXS).

#### X-Ray photoelectron spectroscopy

For identification of surface functional group on the OP and SEP, X-ray photoelectron spectroscopy was performed (Model: AXIS Supra, Kratos Analytical (Shimadzu Group), U.K.) and compared with the original particle (OP). Spectra have also been smoothed and deconvoluted to identify the individual groups in OP and SEP surface.

#### Surface wettability using contact angle

Wetting behavior of OP and SEP surfaces underwater was observed with an optical microscope. In addition, static contact angle (CA) measurement was used to quantify the wettability of OP and SEP, 2 mm glass particles (Indo Glass Beads, India) were used as a substitute of 150–200 μm size particles. In brief, 1 µL droplets of water and LB separately were deposited onto samples by a microsyringe. Images of the liquid-air interface were captured by a digital microscope in drop shape analyzer (DSA, Kruss, Germany). All measurements were averaged over repeated measurements of a sample at room temperature and humidity.

#### Silver loading and Silver leaching

To quantify the silver (Ag^+^**)** loading in the OP^+^ and SEP^+^ particles and Ag^+^ leaching in the purified water, an acid digestion was performed of the supernatant and filtrate collected from the loading and filtration experiments respectively and analysed the amount of silver with Inductive Coupled Plasma Optical Emission Spectrometry (ICP-OES, GF-AA, Perkin- Elmer Analyst 100).

### Preparation of SEP and SEP^+^

All glass particles were cleaned with deionized water and sonicated for 30 min in acetone, followed by drying in hot air oven (EIE, Instrument Pvt. Ltd. India) prior to use. Surface modification of glass particles was performed by refluxing with 4 M NaOH solution for 90 min at 100 °C. The original particle is referred as OP and glass particles treated for 90 min is referred as surface engineered particles (SEP) hereafter. The SEP was then rinsed with de-ionized water until the pH of retaining water becomes neutral (6.8–7.2). Subsequently, the SEP particles were dried overnight in a hot air oven at 65 °C and stored in an airtight container in room conditions. Thereafter, concentration 0.01 mM, 0.03 mM, 0.05 mM, and 0.10 mM silver nitrate (250 ml) was prepared by diluting silver stock solution (1 mM). The substrates both OP and SEP particles (15 gm of each) were separately immersed in an aqueous solution containing 0.01 mM, 0.03 mM, 0.05 mM, and 0.10 mM silver nitrate for 24 hours. They were incubated in a thermostatic shaker (Remi, India) at a speed of 250 rpm in the dark condition at room temperature for 24 h. The substrate containing silver were separated from the mixture and washed with deionized water, and the oven dried for 24 hours at 105 °C. The oven dried samples are collected and stored in airtight containers. The silver ion (Ag^+^) loaded original particles and surface engineered particles are referred to as OP^+^ and SEP^+^ respectively hereafter.

### Bacterial removal, inactivation, and determination of viability

The bacterial removal activity of the OP, SEP, OP^+^, and SEP^+^ particles was tested against a non-pathogenic strain of *Escherichia coli (E. coli)* OP50 (C.elegens Genetics Centre, University of Minnesota, USA), a Gram-negative bacteria. For each experiment, the bacterial culture was grown to OD- 0.40 to 0.50 measured at a wavelength of 600 nm by UV-vis spectrophotometry (Analytic Jena, Germany), which corresponds to 3 × 10^8^ to 4 × 10^8^ cells/ml approximately. In gravity driven filtration experiment, prepared bacterial culture, a model for contaminated water, was passed through columns packed with each type of particle bed (OP, SEP, OP^+^ and SEP^+^ particles) under gravity. A typical experiment involved running 15 ml of the media (called as influent) containing bacteria through the column. Effluent samples were collected in the sterile test tubes and 20 μl of effluent was plated on fresh LB agar plate without any dilution for any viable bacterial growth. Triplicate of experiments were performed for each sample. The influent and effluent samples from each column filters were collected and quantified for *E. coli* contents using a dual beam UV-vis spectrophotometer using LB broth medium as a control. Experimental cell capture efficiency was calculated using the following equation:

4 $$lo{g}_{10}\,remova{l}_{exp}=-lo{g}_{10}\,[\frac{C}{{C}_{0}}]$$

where C_0_ and C are the cell concentration in influent and effluent respectively.

### Cell fixation and SEM

SEM was performed to visualize the changes in the surface morphology of bacterial cells before and after silver loading. For microscopic inspection, after each disinfection test, 100 ml of PBS was run through the filter to clean it and remove any residual bacteria. A more rigorous cleaning was performed prior to further testing using formaldehyde, followed by ethanol. Substrates were then dried and coated with Platinum (Pt) and imaged by FESEM.

## Supplementary information

Movie S1

Movie S2

Supplementary Information

## Data Availability

The final dataset and accompanying material are available on request.
