# Supplementary material for: A Non-electric and Affordable Surface Engineered Particle (SEP) based Point-of-Use (POU) Water Disinfection System
Source: Sci Rep. 2019 Dec 3;9:18245. doi: 10.1038/s41598-019-54602-3 (PMC6890752; doi:10.1038/s41598-019-54602-3)
Supplement: Supplementary file 3 — Supplementary Information [file 41598_2019_54602_MOESM3_ESM.docx]

**A Non-electric and Affordable Surface Engineered Particle (SEP) based Point-of-Use (POU) Water Disinfection System**

Deepa Dixit**^a^**, Virupakshi Soppina^b^, Chinmay Ghoroi**^a^***,

**^a^**DryProTech Lab., Chemical Engineering ,

**^b^**Biological Engineering

Indian Institute of Technology Gandhinagar,

Palaj, Gandhinagar, Gujarat, India-382355

*Corresponding Author: [chinmayg@iitgn.ac.in](mailto:chinmayg@iitgn.ac.in)

Tel: 079-2395-2405

1. **Contact angle Measurement**

LB media droplet contact angle with original particle (OP) - Movie S1

LB media droplet contact angle with surface engineered particle (SEP) - Movie S2
